# Prevalence and comorbidities of autism among children referred to the outpatient clinics for neurodevelopmental disorders

**DOI:** 10.11604/pamj.2016.25.82.4151

**Published:** 2016-10-17

**Authors:** Davin Mbeya Mpaka, Daniel Luwa E-Andjafono Okitundu, Ally Omba Ndjukendi, Adelin Mankubu N’situ, Sebastien Yabassi Kinsala, Joachim Ebwel Mukau, Valentin Malanda Ngoma, Espérance Kashala-Abotnes, Samuel Ma-Miezi-Mampunza, Annick Vogels, Jeans Steyaert

**Affiliations:** 1 Department of psychiatry, Center for Neuro- Psycho- Pathology, School of Medicine, University of Kinshasa, Democratic Republic of the Congo; 2 Department of Neurology, Center for Neuro- Psycho- Pathology, School of Medicine, University of Kinshasa, Democratic Republic of the Congo; 3 National University of Pedagogy and Center for Assessment and Intervention for Children with mental Handicap and/or Autism, Democratic Republic of the Congo; 4 Centre for International Health, Department of Global Public Health and Primary Care, University of Bergen, Bergen, Norway; 5 Katholieke Universiteit Leuven (KUL)

**Keywords:** ASD, comorbidity, intellectual disability, epilepsy, neuro-developmental disorders, prevalence, Kinshasa/ Democratic Republic of the Congo

## Abstract

**Introduction:**

Autism spectrum disorders (ASD) is a neurodevelopmental disorder that has been rarely diagnosed in Sub-Saharan Africa. Although a proportion of children do present features of ASD in the Democratic Republic of Congo (DRC), little is known about it prevalence. Often, the co-morbidities constitute the upfront symptoms and therefore may it recognition and management difficult, aggravating as such the prognosis. The present study therefore aimed at studying the clinical profile of autism spectrum disorder (ASD) and the associated morbidities among children and adolescents in outpatient clinics in Kinshasa, the Democratic Republic of Congo.

**Methods:**

We conducted a cross sectional study in the three outpatients centers receiving patients referred for neurodevelopmental disorders in Kinshasa, DRC, from June 2008 to June 2010. A total of 450 subjects aged from 1-18 years old were referred and included in the study. The clinical diagnosis for ASD was made using the DSM-IV-R and the ADIR. Co-morbidities were identified using DSM-IV-R criteria together with an extensive clinical interview and observation. All patients were subject to an intellectual quotient evaluation and an electroencephalogram reporting.

**Results:**

Of the 450 subjects referred, 120 (29.3%) received the diagnosis of ASD, with boys outnumbering girls (OR 3:1. The mean age was 7.9 years (SD 3.4) (p< 0.001). Intellectual disability (75.83 %) and epilepsy (72.50%) were the main co-morbidities significantly associated with autism (p< 0.001). It was also found that co-morbidities were most frequent in subjects with an IQ<70 (p=0.05).

**Conclusion:**

ASD is frequent among patients referred for neurodevelopmental disorders in the three outpatients’ centers for neurodevelopmental disorders in Kinshasa. Males seem to be more affected than female. The main co-morbidities were epilepsy and intellectual disabilities. Our findings suggest that it is important to screen for ASD and co-morbidities among all subjects referred for neurodevelopmental disorders and to undertake survey on ASD in various structures of rejected children from the society in Kinshasa DRC. This will help to identify and manage ASD and associated co-morbidities at an early stage for a better prognosis.

## Introduction

Autism is a severe neurodevelopmental disorder with prevalence varies from 0.6 to 1 % [1–3]. Autism is at least three times more common in males [1, 4, 5]. It is characterized by disorders in social interactions, communication and behavior, which must appear under 3 years [1]. It is also characterized by clinic, etiologic and therapeutic heterogeneities [6, 7] and by multiple neurologic, psychiatric and somatic comorbidities [6, 8–11]. Autism is in comorbidity relationship with epilepsy in 30% [12], with intellectual disability in 50-80% [11] and with attention deficit hyperactivity disorder (ADHD) in 20 to 85 % [3, 11]. Other disorders such as X-fragile, Rett syndrome, tuberous sclerosis are also described [3, 11]. Intellectual disability, epilepsy and ADHD can share a common neurobiological basis [3, 13] and are factors of poor prognosis of autism [3, 14]. Comorbidities are the main reasons for referral to outpatient clinics and admission to hospitals. They mask and delay the diagnosis and are the cause of inadequate therapies [6, 15, 16]. That is why Gadow, Devincent, & Schneider, 2008 [17] strongly recommend looking at the presence of comorbidities before starting any treatment of autism.

There are little data on the prevalence of autism in Sub-Saharan Africa [18]. Autism is rarely diagnosed in these countries because it is confused with development, neurological or psychiatric disorders, with dumbness and with deafness [6, 19]. In addition, autism is treated independently from its comorbidities in specialized neurodevelopmental disorder treatment centers and in general medical settings [19]. Consequently its magnitude is hidden which does not help for planned cares of children suffering from autism. In the study on diagnosis and interventions in children with autism, intellectual disability, deafness and other handicaps, Mukau [19] ; did not do a systematic research of comorbidities of autism as Gadow, Devincent, & Schneider [17] recommended. Consequently, the aim of the current study was first to determine the prevalence of autism among children in outpatient clinics of neurodevelopmental disorders in Kinshasa; and second to identify the main comorbidities of autism in this population.

## Methods

### Sample

From June 2008 to June 2010, 405 children aged 1 from to 18 years were referred to 3 Centers taking care of children and teenagers affected by neurodevelopmental disorders. A research team of 3 psychiatrists, 1 neurologist, 1 ENT-specialist, 1 neuropediatrician, 1 otolaryngologist and 2 clinical psychologists was formed to diagnose autism. Out of the 405 children referred to outpatient clinics of neurodevelopment disorders, 213 were diagnosed with Pervasive developmental disorder (PDD) according to DSM-IV-R criteria [1] and to Scale of Pervasive Disorder in Mentally Retarded Persons (PDD-MRS) [20], The French adapted version of Dutch original version [19]. Autism Diagnosis Interview Revised (ADIR) [21]; was used to diagnose autism. Children without autism on ADIR (n=56) and those whose parents or tutors were not present during interviews (n=20) or refused ADIR interview (n=7) and those who were under 3 years at the end of the study (n=10) were excluded.

### Procedures and techniques of data collection

We looked at socio-demographic characteristics and co-morbidities in children diagnosed with autism. The diagnosis of comorbidities was done by medical history carried out with children’s parents, clinic neuropsychiatric observation, DSM-IV-R criteria [**1**], audiometric, assessment of Intellectual Quotient of performance (IQ) and electroencephalography (EEG). The audiometric assessment was done by the ENT-specialist in order to exclude hearing impairments, which is often misdiagnosed in children with ASD and because children with autism seem to be deaf without being like it and deafness may be a comorbidity of autism. Assessment of intellectual level through the Wechsler Performance Test (WISC-R) [22] was done by the clinical psychologist. Intellectual disability was equivalent to IQ < 70 in Wechsler Performance Test (WISC-R) [22]. The results of routine electro-encephalogram recorded among children with autism were classified in normal EEG (clear EEG), or pathological EEG. Pathological EEG were abnormal EEG with slow activities related to age, worse stopping visual reaction, irregular rhythm and /or the presence of epileptic patterns, in accordance with the conclusions of EEG protocols done in the EEG laboratory of the University of Kinshasa. The diagnosis of epilepsy was based on parent’s report of convulsive seizures, complete neurological examination and on abnormal electrical activities in intercritic routine EEG records. Neuropsychiatric examination and DSM-IV-R criteria [1] were used to diagnose psychiatric disorders, intellectual disability, schizophrenia, anxiety, depression and ADHD. Only cases of hyperactive children with attention deficit above 7 years were considered as ADHD. The diagnosis of neurological disorders (cerebral palsy and other encephalopathy) was done through performed by child’s medical history and complete physical and neurologic examination.

### Statistical analyses

Database and acquisition were performed by the Sphinx Plus2 software then exported on Excel 2007 for finally encoding and cleaning towards the Statistical Package for Social Sciences (SPSS 17.0) software for statistical analyses. Comparison of observed numbers of observations was done by using the binominal test and Pearson's chi-squared test. We used Spearman’s correlation to display relationship between age and IQ, Kruskal-Wallis to relate age to comorbidities and analysis of covariance for the link between the number of comorbidities and IQ scores adjusted to age. The threshold of significance was p < 0.05.

## Results

### Profile of disorders in children referred to outpatient clinics for neurodevelopmental disorders

In a comparative view of autism with other development disorders, Table 1 shows how children were divided in relation with gender and disorder among the 405 targeted children (mean age 6.91 ± 4.3 years and sex-ratio M: F 2.1:1). The data in this chart are given in terms of relative frequency and percentage. At least one child out of two treated in three specialized neurodevelopmental disorders treatment centers (52.59%; p = 0.003) had pervasive development disorders (PDD) (Table 1). The children of the population study (n=405) had a mean age of 6.91 (±4.3) years. The sex ratio M: F was 2. 1:1. Table 1 also shows the distribution by sex of all children and a comparative view of various pathologies. The high frequency of male was found mainly among children with Hyperactivity without or with attention disorder (M 12.75 % Versus F 3.8 %; p = 0.001), autism (M 35. 80 % Versus F 20.37%; p = 0.001) and PDD (58.88% Versus F43.21 %; p= 0.003).

**Table 1:** Disorders in 405 the children referred to the outpatient clinic of neurodevelopmental disorders according to gender

Disorders	Total	Gender		P
	n=405	M=243	F=162	
**Neurological disorders**				
Epilepsies	269 (66.42)	166 (68.31)	103(63.58)	>0.05
Non-specified Encephalopathy	66 (16.29)	42 (17.28)	24 (14.81)	> 0.05
Cerebral palsy	178 (43.95)	102(41.97)	76(46.91)	> 0.05
**Psychiatric disorders**				
PDD	213 (52.59)	143(58.88)	70 (43.21)	0.003
Autism	120 (29.62)	87(35.80)	33 (20.37)	0.001
Other PDD	93 (22.96)	56(23.04)	37(22.83)	0.002
Hyperactivity or ADHD	36 (08.88)	31 (12.75)	5(3.08)	0.001
Schizophrenia	22 (05.43)	12 (4.93)	10 (0.17)	> 0.05
Depression	14 (03.43)	4 (1.64)	10 (6.17)	0.016
Anxiety	14 (03.43)	4 (1.64)	10(6.17)	0.016
**Cognitive disorder**				
Development or intellectual disability	134 (33.09)	81 (33.33)	53 (32.71)	> 0.05
Others	20 (04.93)	11 (4.52)	9 (5.55)	> 0.05

### General characteristics of autistic children

Autism was found among 29.26 % of children with development disorders (120/450) and affected 87 Males (72.50%) and 33 Females (27.50%), with a sex-ratio M: F of 2.6: 1. Children with autism ranged from 3 to 17 years, with a mean age (± standard deviation) of 7.9 ± 3.4 years, a median of 7 years and a mode of 5 years. Males were younger than Females (8 ± 3 years versus 9 ± 4 years; p=0.004). During the diagnosis, children from 3 to 6 years (40. 83%) and from 7 to 12 years (42. 17%) were in majority (Table 2).

**Table 2:** Age groups gender in autism

Group of age (years)	Total N= 120	Gender (%)		P
		M	F	
		87(72.50)	33(27.50%)	
3 – 6	49(40.83%)	39(44. 83%)	10 (30.30%)	> 0.05
7 – 12	53(42.17%)	39(44.83%)	14 (42.42%)	
13 – 17	18(15.00 %)	9(10.34%)	9 (27.27%)	

### Autism and co-morbidities

**Figure 1:**
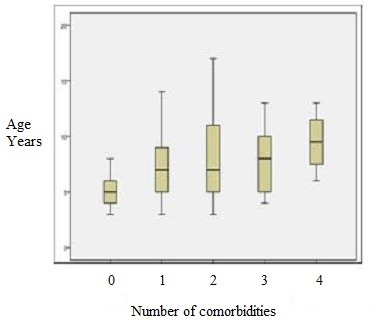
Link between age and comorbidity children with great number of comorbidities were older than the others

**Figure 2:**
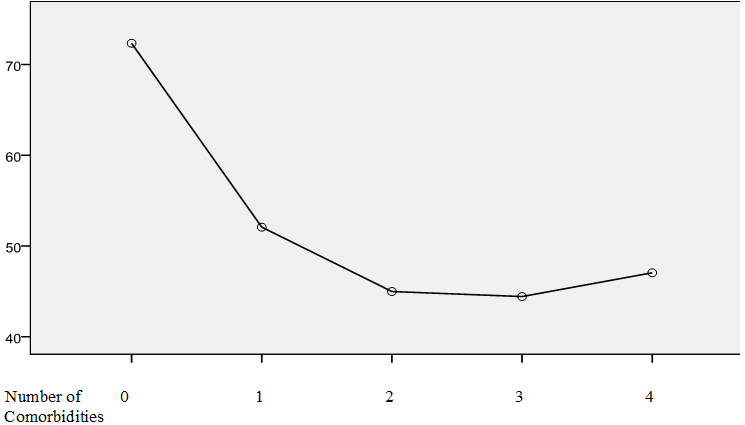
Decrease of IQ according to the number of comorbidities

Cognitive disorders or intellectual disability according to Performance IQ (75.83 %) and epilepsy (72.50%) were the main comorbidities significantly associated with autism (p<.001) (Table 3). In total, 93.33% of cases had 1 to 4 comobidities and the EEG showed abnormal activities even in cases without comorbidities (Table 4).Table 4 also shows that 6.67% (8/120) of children with autism were without comorbidities, and had an IQ more than 70 and normal audiometry in 100% (8/8) and 62.50% (5/8) of them had abnormal EEG. The frequencies of abnormal EEG and audiometric varied with the presence of comorbidities. The frequency of IQ < 70 or of cognitive disorders was high relation with the number of comorbidities, it reached 100% when the comorbidities are 3 or 4 (p=0,014) (Table 4). There was a significant relationship between age and number of comorbidities in autism, children with high number of comorbidities were older than those with low number or without comorbidities (K (4)=10.222, p=.037) (Figure 1). The correlation between age and IQ was less with meaningful (r = -. 21; p = .010). We noticed a significant effect of number of comorbidities on IQ scores after controlling age (F (4, 114) = 6.018, p < .001). The IQ adjusted to the age (age=7.93years) decreased in relation with the number of co morbidities in autistic children (Figure 2).

**Table 3:** Comorbidity with autism

Comorbidities	Associated of Autism		P
	Yes (n %)	No (n %)	
**Cognitive disorders**			
Development or intellectual disability	91 (75.83)	29 (24.17)	< 0.001
**Neurological disorders**			
Epilepsy	87 (72.50)	33 (27.50)	< 0.001
Cerebral palsy	08 (06.67)	112 (93.33)	< 0.001
Encephalopathy	16 (13.33)	104 (86.67)	< 0.001
**psychiatric disorders**			
ADHD	17 (14.17)	103 (85.83)	< 0.001
Schizophrenia	14 (11.67)	106 (88.33)	< 0.001

**Table 4:** Para clinic assessments and co morbidities number in autism

Assessments			Number of co morbidities					P
			0	1	2	3	4	
		n (%)	8 (6.67)	37(30.83)	53(44.17)	14(11.77)	8 (6.67)	
IQ	> 70	29(24.17)	8 (100)	14(37.83)	7 (13.21)	0 (0.00)	0 (0.00)	<0.001 0.014 > 0.05
	< 70	91 (75.83)	0 (0.00)	23(62.16)	46(86.79)	14 (100)	8 (100)	
EEG	Normal	26 (21.17)	3(37.50)	14(37.83)	8 (15.09)	0 (0.00)	1(12.50)	
	Abnormal	94 (78.33)	5(62.50)	23(62.16)	45(84.91)	14 (100)	7(87.50)	
Audiometry	Normal	89(74.17)	8 (100)	29(78.38)	35(66.00)	11 (7.57)	6 75.00)	
	Abnormal	31 (25.83)	0(0.00)	8 (21.62)	18(34.00)	3 (21.43)	2(25.00)	

## Discussion

This study aims at determining the prevalence and co morbidities of Autism among children referred to the outpatient clinic of Neurodevelopmental disorders. There was male predominance in autism which is consistent with data reported in Western countries [4] as well as in Africa [8, 18, 19]. The prevalence of autism was 29.62%, and it is similar to the data described in a previous study in Northern Africa. Seil Eldein et al. [23] described a prevalence of 33.6% in Egypt and of 11.5% in Tunisia. Mukau [19] found a rate of 53%, the latter high prevalence is equivalent to that of 52.59% of Pervasive Development Disorders (ASD: Autism Spectrum Disorder) found in our study. This could be explained by the fact that Mukau [19] in his series, recruited children with autism among children with intellectual disability. Intellectual disability (ID) is known to be associated with ASD; 25-40% of the children with intellectual disability are diagnosed with ASD [24, 25]. There is a great difference in comparison with general population in which 1 to 3% has ID [4]

In our study 75.83% of the children diagnosed with autism had intellectual disability; this corroborates previous studies reporting a prevalence of 50 to 80% [1, 4, 6, 19]. The frequency of epilepsy, 72.50% in our study, is high compared to the frequencies depicted in Western studies which tend to be 46% [4, 7, 10, 12, 26]. This high frequency of epilepsy in our series can be explained by early cerebral morbidity known to be prevalent in developing countries [11, 27, 28]. The significant relationships between autism, intellectual disability, and epilepsy which are the three main neurodevelopmental disorders in our study prove the damaging role of early cerebral morbidity. The harmful role of these factors is known to be involved in the pathogenesis of neurodevelopment disorders [13, 29, 30]. In addition, intellectual disability and epilepsy are known to be predictor factors of worse prognosis, morbidity and mortality of autism [14].

Our study showed on the one hand, the increase of frequency of intellectual disability and the decrease of IQ with co morbidities, and one the other hand the high frequency of pathological EEG even in the case of autism without co morbidities. Mukau [19] and Rommelse et al. [3] assumes that some co morbidities can be responsible for weak IQ but Pry R et al. [31] think that the socio-cognitive factors can affect the IQ and improve it in the growing age. We think that EEG perturbations in autism without co morbidities are an argument for the neurobiological bases of this disease [3, 6, 7]. These data suggest the need of conducting a genetic study on autism co morbidity in our area. The findings of this study will enable to make a difference between genetic and environmental factors in order to direct the genetic counsels and minimize the impact of culture on the origin of autism [18]. The proportion of pathological audiometric test (25.83 %) was high compared to the data reported in the literature (5.9 to 17%) [32, 33]. This high proportion may be due to the early cerebral morbidity in African areas.

ADHD is frequently associated with autism. The diagnosis of this disorder is difficult to make when it is associated with autism. The frequency of 14.5% observed in our work, are lower than those of Leyfer et al. [10] and Gillberg et al [34] who described respectively 55% and 65 to 80% of cases. This may be explained by socio-demographic characteristics: almost 41% of children in our series were in the pre-schooling. The proportion of autistic children with schizophrenia was 11.6% in our work in contrast to data from Mouridsen, Rich & Isager [35]; who reported a prevalence of 30% for schizophrenia in autistic teenagers. The low number of teenager in our sample (15%) can explain our low ratio of schizophrenia in our sample, the age at onset of schizophrenia is usually adolescence.

## Conclusion

Autism is frequent among children referred for the outpatient clinic of neurodevelopment disorders. Males are more frequently affected than females. Frequent comorbidities are intellectual disability and epilepsy. The number of co morbidities ranged from 1 to 4 in 93. 3% and was inversely associated with intelligence IQ. A pathological EEG was frequently founded even in the case of absence of any co morbidity. The assessment of children referred with the outpatient clinic of neurodevelopment disorders should include a diagnostic assessment of autism.

### What is known about this topic

- Autism spectrum disorders (ASD) has been rarely diagnosed in Democratic Republic of Congo (DRC). The prevalence of ASD is unknown;
- ASD children are mixed up with those victims of other neurodevelopmental disorders, considered as witchcraft and bareness, consequently rejected from the society;
- The presence of associated co-morbidities complicates the assessment, the prognosis and management.

### What this study adds

- ASD are frequent in neurodevelopmental centers and among rejected children;
- The number of co-morbidities affect the child’s QI, the main of them, epilepsy and intellectual disabilities have a risen rate when comparing with other countries;
- Finally our study recommend to undertake survey on ASD specifically in others structures of rejected children from the society in Kinshasa DRC.
